# Maraviroc Failed to Control Progressive Multifocal Leukoencephalopathy-Associated IRIS in a Patient with Advanced HIV Infection

**DOI:** 10.1155/2014/381480

**Published:** 2014-12-23

**Authors:** Mónica Rodríguez, Fernando Antonio Silva-Sánchez, César Luna-Rivero, Ricardo Vega-Barrientos, Claudia Alvarado-de la Barrera, Gustavo Reyes-Terán

**Affiliations:** ^1^Centro de Investigación en Enfermedades Infecciosas, Instituto Nacional de Enfermedades Respiratorias Ismael Cosío Villegas, Calzada de Tlalpan 4502, Colonia Sección XVI, 14080 México, DF, Mexico; ^2^Servicio de Patología, Instituto Nacional de Enfermedades Respiratorias Ismael Cosío Villegas, 14080 México, DF, Mexico; ^3^Servicio Clínico 4, Instituto Nacional de Enfermedades Respiratorias Ismael Cosío Villegas, 14080 México, DF, Mexico

## Abstract

Due to the lack of therapeutic options for patients with progressive multifocal leukoencephalopathy-associated immune reconstitution inflammatory syndrome (PML-associated IRIS), maraviroc has generated expectations among the medical community. However, we report a patient with advanced HIV infection, who developed PML-associated IRIS and had a fatal outcome despite the addition of maraviroc to suppressive ART. Future studies are required to define the therapeutic role of maraviroc in PML-associated IRIS and differentiate individuals who may benefit from maraviroc from those who may develop neurological deterioration.

## 1. Introduction

Progressive multifocal leukoencephalopathy (PML) remains an incurable and often fatal disease, for which HIV infection is the most frequent immunodeficiency setting [1]. There is no specific treatment, but reversal of immunosuppression by antiretroviral therapy (ART) leads to clinical stabilization in 50–60% of PML patients [2]. Considering that beneficial effects of the CCR5 antagonist maraviroc were reported in two patients with PML-associated immune reconstitution inflammatory syndrome (IRIS)—the first with HIV infection [3] and the second with multiple sclerosis [4]—here we describe a patient with advanced HIV infection, who developed PML-associated IRIS and had a fatal outcome despite the addition of maraviroc to suppressive ART.

## 2. Case Presentation

**Figure 1:**
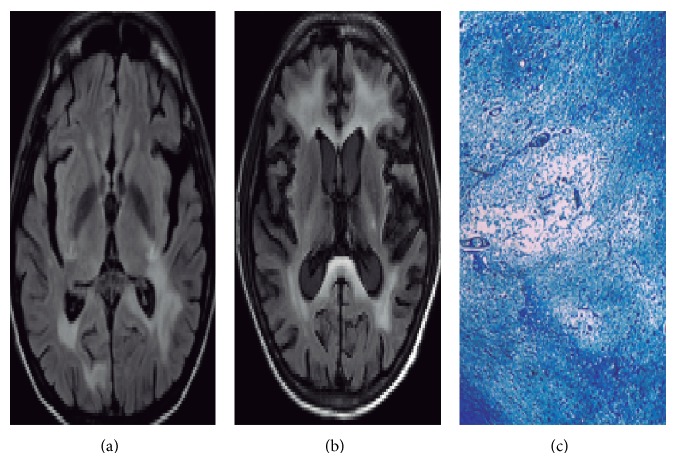
Sequential MRI of the brain and myelin stain. Axial FLAIR images show hyperintensity in patches of subcortical white matter localized in parietal and occipital lobes (a). Premortem axial FLAIR images show increased lesions in bilateral frontal lobes without mass effect after treatment with maraviroc (b). Kluver-Barrera stain (10x) for myelin revealed brainstem zones of demyelination and enlarged oligodendrocytes with hyperchromatic nuclei and bizarre astrocytes (c).

A 55-year-old Mexican individual presented to our institution with a 3-year history of HIV-1 infection and virologic failure after ART interruption (125697 HIV RNA copies/mL, 5.1 log_10_). Patient informed consent for tests performed for clinical purposes using routine techniques was obtained. Three months before admission, he had initiated ART consisting of abacavir, tenofovir, emtricitabine, and atazanavir/ritonavir. After 1 month on ART, he suffered from fever and productive cough. One week before admission, he also had diarrhea and abdominal pain. On admission, he had a CD4 T cell count of 7 cells/*μ*L and <40 HIV RNA copies/mL, and the neurological examination showed no abnormalities. Chest computed tomography (CT) revealed bronchiectasis and consolidation at right basal segments, pericardial and bilateral pleural effusion, hepatosplenomegaly, biliary lithiasis, and free fluid in the abdominal cavity. Cultures of bronchoalveolar lavage, blood, rectal biopsy, and feces were positive for* Mycobacterium avium* complex (MAC). MAC-associated IRIS was diagnosed [5], and oral ethambutol, clarithromycin, and azithromycin were prescribed. During hospitalization he developed neurological impairments including visual anosognosia (Anton-Babinski syndrome), afferent pupillary defect, apraxia (of gait, dressing, and eating), right hemiparesis, generalized tonic-clonic seizures, and cognitive deterioration. The CT and magnetic resonance imaging (MRI) of the brain revealed hypointensity on T1 and hyperintensity on T2 and FLAIR images in the subcortical white matter. The lesions had a bilateral, parietal-temporal, occipital, and internal capsule distribution characteristic of PML (Figure 1(a)). MR spectroscopy revealed reduced N-acetylaspartate, increased lipids, and generalized cortical and subcortical atrophy. Cerebrospinal fluid (CSF) was acellular; biochemistry was normal; and cultures were negative for mycobacterial, fungal, parasitic, and conventional bacterial agents. HIV RNA was undetectable in CSF, but JC virus DNA was 822.5 copies/mL (2.91 log_10_). The patient was diagnosed with PML-associated IRIS [5], so maraviroc 300 mg twice daily and dexamethasone 8 mg every 8 hours for 3 days were added to ART. The patient had a rapid clinical deterioration and died 21 days after maraviroc initiation with a JC virus DNA load of 612.5 copies/mL (2.78 log_10_). Premortem MRI revealed lesions in subcortical white matter (Figure 1(b)). Autopsy findings included zones of demyelination, enlarged oligodendrocytes with hyperchromatic nuclei and bizarre astrocytes, and brainstem lesions (particularly in medulla oblongata) (Figure 1(c)). Despite the fact that autopsy revealed the presence of MAC in lungs, kidney, and other organs, there was no evidence of septic shock or multiple organic failure. Instead, brainstem demyelination was considered related to subsequent respiratory failure and death.

## 3. Discussion

In contrast with the beneficial effects of maraviroc previously reported in two patients with PML-associated IRIS [3, 4], our patient experienced neurological deterioration. One possible explanation for these contrasting results is that the efficacy of maraviroc could be affected by the degree of immunocompromise and the stage of PML disease. That is, maraviroc efficacy may be probably higher if administered on earlier stages of HIV and JC virus disease. Alternatively, the clinical deterioration of our patient may be related to the immunomodulatory properties of maraviroc. By blocking the CCR5 chemokine receptor, maraviroc inhibits innate and adaptive immune responses, which may actually aggravate the immunocompromised condition of HIV-infected patients. In fact, a complete loss-of-function mutation in CCR5 is associated with symptomatic neuroinflammatory disease for West Nile and tick-borne encephalitis flavivirus infections [6, 7]. By consequence, the possibility that administration of CCR5 inhibitors may increase the risk of opportunistic infections and malignancies in HIV-infected individuals should be considered [8]. In addition, corticosteroids are commonly used in the management of PML-associated IRIS, but their potentially negative effects on the JC virus-specific response may have contributed adversely [9].

A higher proportion of CD8+ T cells coexpressing CCR5 and CXCR3 was found in CSF of patients with cryptococcosis-associated IRIS compared with blood at ART initiation [10]. Thus, CCR5+ T cells have been indirectly implicated in IRIS pathophysiology, and maraviroc is expected to have a beneficial effect on different clinical manifestations of IRIS by interfering with the traffic of effector lymphocytes to sites of opportunistic infection. However, since patients with IRIS usually have low CD4 T cell counts, aggravation of immunodeficiency may have fatal consequences in this particular group. Therefore, close clinical and immunologic monitoring of clinical trials exploring the efficacy of maraviroc in patients with IRIS is mandatory. Future studies are required for identification of patients with IRIS who may benefit from maraviroc.
